# Anxiety and depression among caregivers of pediatric patients with tic disorder in western China: A cross-sectional study

**DOI:** 10.1371/journal.pone.0289381

**Published:** 2023-07-28

**Authors:** Zheng Liu, Chunsong Yang, Dan Yu, Linan Zeng, Zhi-Jun Jia, Guo Cheng, Lingli Zhang

**Affiliations:** 1 Department of Pharmacy, West China Second University Hospital, Sichuan University, Chengdu, China; 2 Evidence-Based Pharmacy Center, West China Second University Hospital, Sichuan University, Chengdu, China; 3 NMPA Key Laboratory for Technical Research on Drug Products In Vitro and In Vivo Correlation, Chengdu, China; 4 Key Laboratory of Birth Defects and Related Diseases of Women and Children, Sichuan University, Ministry of Education, Chengdu, China; 5 West China School of Medicine, Sichuan University, Chengdu, China; 6 Department of Children’s Genetic Endocrinology and Metabolism, West China Second University Hospital, Sichuan University, Chengdu, China; 7 West China School of Pharmacy, Sichuan University, Chengdu, China; 8 Department of Pediatrics, West China Second University Hospital, Sichuan University, Chengdu, China; 9 Laboratory of Molecular Translational Medicine, Center for Translational Medicine, Sichuan University, Chengdu, China; 10 Chinese Evidence-based Medicine Center, West China Hospital, Sichuan University, Chengdu, China; Research, Training and Management International, BANGLADESH

## Abstract

**Background:**

Caregivers of pediatric patients with tic disorders (TD) are at high risk for anxiety and depression, but the situation of this disorder was rarely reported based on the Chinese population. The purpose of this study was to investigate the prevalence and potential contributing factors of anxiety and depression among caregivers of Chinese pediatric patients with TD.

**Methods:**

A cross-sectional study was carried out on caregivers of pediatric patients with TD at a women’s and children’s hospital in western China from January to June 2020. A structured questionnaire was designed to collect data, including socio-demographic information, disease and medication status, family situation and social relationship, cognition and attitude towards TD and treatment. Anxiety and depression were assessed using the self-rating anxiety scale (SAS) and self-rating depression scale (SDS), respectively. The univariate analysis and multivariate logistic regression were used to analyze the cross-sectional data.

**Results:**

A total of 318 participants were included in this study, with a response rate of 89.58% (318/355). The average age of pediatric patients with TD was 8.38 ± 2.54 years, and 78.30% (249/318) of caregivers were aged between 30–50 years old. Overall, 14.78% (47/318) of caregivers presented the symptom of anxiety, with a mean SAS score of 54.81±5.26, and 19.81% (63/318) of caregivers presented the symptom of depression, with a mean SDS score of 59.64±5.83. Logistic regression analysis revealed that the common family relationship (OR = 2.512, p = 0.024), and pediatric patients with unharmonious social relationships (OR = 5.759, p = 0.043) and with introverted personality (OR = 2.402, p = 0.023) were significantly associated with anxiety in caregivers of pediatric patients with TD, as well as the single-parent family (OR = 4.805, p = 0.011), mistaken cognition of TD (OR = 0.357, p = 0.031), and pediatric patients with fewer friends (OR = 3.377, p = 0.006) were significantly associated with depression.

**Conclusions:**

Anxiety and depression are prevalent among caregivers of TD pediatric patients, which brings up the importance of psychiatric support for this group. Longitudinal studies need to be conducted to further confirm the causality before interventions to improve mental health are developed.

## Introduction

Tic disorder (TD) is a neurodevelopmental disease defined as sudden, rapid, recurrent, and nonrhythmic movements or vocalizations with onset in childhood and adolescence [1], including provisional tic disorder (PTD), chronic tic disorder (CTD), and Tourette syndrome (TS), with a global prevalence of 2.99%, 1.61%, and 0.77%, respectively [2]. TD is likewise a common neuropsychiatric disorder in China that affects 6.1% of the pediatric population [3]. In addition to frequent tics, about half of children with TD suffer from comorbidities, including attention deficit hyperactivity disorder (ADHD), obsessive-compulsive disorder (OCD), anxiety, depression, and sleep disorders [4]. Due to the lifelong disorders of TD that broadly impact patients’ quality of life throughout their duration, it has detrimental effects on the emotional, social, and physical well-being that persist into adulthood, and presents family and patients with unique challenges [5].

The pathogenesis of TD is complex that could be the combined result of genetic, immunological, psychological, and environmental factors [4]. The caregivers of pediatric patients with TD, as one of the environmental factors, play an important role in the management of the disease. However, the parenting stress of a child with TD, including long-term care, lack of support, and fears of missing work, takes an emotional and physical toll on the caregiver as well [6]. Besides, it seems more likely that those parents experience extensive social stress and increasing mental burden due to the potentially stigmatizing nature of symptoms than parents of children with chronic illness [7], especially when children suffering from TD with comorbidities [8,9]. And previous research indicated that these perceived stresses were highly correlated with their anxiety and depression [10].

While on the other hand, the health state of parents can, in turn, affect the condition and psychosocial functioning of pediatric patients [11–13]. Thus, medical education and psychological support for patients and caregivers are generally regarded as the first step in the intervention of TD, which can improve parenting behavior and mental health, and contribute to the tics subside for patients [4,14]. A better understanding of anxiety and depression among caregivers will help to explore the starting points for psychological interventions and provide available support for caregivers. The mental situation of caregivers having a child with TD, however, was rarely reported in previous studies. Therefore, the present study aimed to investigate the prevalence of anxiety and depression among caregivers of pediatric patients with TD and identify the potential contributing factors associated with anxiety and depression in caregivers.

## Materials and methods

### Study design and setting

A cross-sectional study using a questionnaire was conducted among caregivers of patients from the pediatric neurology department of West China Second Hospital, Sichuan University, the largest women’s and children’s hospital located in western China. This study was carried out from January to June 2020. A questionnaire was designed and pretested by trained investigators to collect data based on the published literature and expert opinions.

### Population and sample size

A consecutive sampling technique was adopted to select eligible participants based on the following inclusion criteria: (i) caregivers of patients with a definitive diagnosis of TD according to the Diagnostic and Statistical Manual of Mental Disorders, Fifth Edition (DSM-V); (ii) caregivers of patients aged less than 18 years; (iii) voluntary to participate in this study and provide informed consent. The exclusion criteria were: (i) other chronic diseases in the pediatric patients that could potentially affect the emotion of caregivers (i.e., congenital heart disease, epilepsy, malignancy); (ii) those who were unable to understand or fill out the questionnaire.

Based on a similar study [15], the sample size was calculated using a formula for single population proportion: n  =  (Z_α/2_)^2^×p× (1−p)/δ^2^, considering a p-value of 26%, 95% confidence level, 5% margin of error, and 20% non-response rate, the final sample size was 355.

### Data collection and instruments

The data were collected using a pretested questionnaire which consists of demographic characteristics, a tic severity assessment tool, and an anxiety and depression screening tool.

The demographic characteristics of participants were collected in two sections. The first section focused on pediatric patients with TD, including: (i) basic information: gender, age, height, weight, and race; (ii) disease status: onset age, times of visit the hospital, type of TD, tic symptom, comorbidity; (iii) medication information: type of medication, course, adverse reaction; (iv) social relationship and psychological quality. The second section focused on the caregivers, including: (i) socio-demographic characteristics: gender, age, job, educational background, and relationship with the child; (ii) family situation: residence, family structure, relationship, circumstance, and medical expenditure; (iii) cognition and attitude towards TD and treatment. Among the above, basic information, disease status, and medication of pediatric patients were checked and verified with medical records from the hospital information system. There was no access to information for researchers that could identify individual participants during or after data collection.

Tic severity was assessed by the Yale global tic severity scale (YGTSS), a semi-structured scale developed for evaluating the tics observed within the previous week and rated by a clinician or trained investigator [16]. The five dimensions included in the YGTSS are number, frequency, intensity, complexity, and interference. The global YGTSS score (range: 0–100) is derived from the summation of the tic severity rating score (range = 0–50, including motor tics ranging = 0–25 and vocal tics ranging = 0–25) and the impairment rating score (range = 0–50), with excellent reliability and validity for assessing children and adolescents with TD [17,18]. Tic severity is finally graded into mild (global score < 25), moderate (25–50), and severe (> 50).

Anxiety and depression were measured by the self-rating anxiety scale (SAS) and self-rating depression scale (SDS), respectively [19,20]. The two self-reporting tools have been widely used in China with good internal consistency (SAS: α  =  0.93; SDS: α  =  0.86) and test-retest reliability (SAS: r  =  0.77; SDS: r  =  0.82) [21]. Both SAS and SDS consist of 20 items with a 4-point scoring system, of which 1 point means “no or a little of the time,” and 4 points mean “most or all of the time”. The total score of 20 items is multiplied by 1.25 to produce the standard score. The standard score of SAS ≥ 50 is diagnosed with anxiety and graded into mild (50–59), moderate (60–69), and severe (≥70), while the standard score of SDS ≥ 53 is diagnosed with depression and graded into mild (53–62), moderate (63–72), and severe (≥73).

### Data analysis

Statistical analyses were performed using IBM SPSS Statistics, version 26.0 (IBM Corp., Armonk, NY, United States). Quantitative variables were expressed as mean and standard deviation (Mean ± SD), and qualitative variables as frequency and percentage (n, %). Normally distributed data were analyzed using the variance analysis and the non-normally distributed data were analyzed using the Mann-Whitney U test to compare quantitative variables between groups. While the qualitative variables were analyzed using the chi-squared test or Fisher’s exact test as appropriate. Following the univariate analysis, the factors with *p* ≤ 0.05 were subjected to the multivariate logistic regression model. Multivariate analysis was adopted to examine the relative contribution of potential factors of anxiety and depression. A *p* ≤ 0.05 was considered to be statistically significant.

### Ethical considerations

The ethics committee of West China Second University Hospital, Sichuan University approved this study (No.2019[8]), and written informed consents were obtained from participants and the parents or guardians of the enrolled pediatric patients.

## Results

### Characteristics of participants

A total of 355 caregivers were eligible and invited to participate in the study, of which 318 completed all questionnaires (89.58% response rate). The pediatric patients with TD were aged between 0.3 and 17.5 years, with a mean age of 8.38 ± 2.54 years, and the onset age of the disease ranged from 0.7 to 13.0 years with a mean of 5.82 ± 2.10 years, among which 63.84% (203/318) of pediatric patients had comorbidities. The proportion of those with mild, moderate, and severe tic symptoms in the study group, respectively, were 80.19% (255/318), 16.67% (53/318), and 63.84% (10/318). The majority of caregivers were aged between 30–50 years old (78.30%, 249/318). Most of them were married (95.91%, 305/318) and completed the survey as parents of pediatric patients (79.25%, 252/318). The demographic characteristics of the participants are shown in Table 1.

**Table 1:** The demographic characteristics of the participants.

Variables	Total (n = 318)	Anxiety		*p*	Depression		*p*
		Yes (n = 47)	No (n = 271)		Yes (n = 63)	No (n = 255)	
**Pediatric patients**							
Age (year), Mean ± SD	8.38 ± 2.54	8.50 ± 2.50	8.35 ± 2.55	0.716	8.24 ± 2.43	8.41 ± 2.56	0.644
Gender, n (%)				0.858			1.000
Boy	240 (75.47)	36 (76.60)	204 (75.28)		48 (76.19)	192 (75.29)	
Girl	78 (24.53)	11 (23.40)	67 (24.72)		15 (23.81)	63 (24.71)	
Height (cm), Mean ± SD	126.07 ± 16.37	124.76 ± 16.27	126.30 ± 16.41	0.662	124.79 ± 15.08	126.39 ± 16.69	0.528
Weight (kg), Mean ± SD	29.49 ± 11.05	29.34 ± 10.80	29.52 ± 11.11	0.993	29.24 ± 11.95	29.56 ± 10.84	0.589
Race, n (%)				1.000			1.000
Han	307 (96.54)	46 (97.87)	261 (96.31)		61 (96.83)	246 (96.47)	
Non-Han	11 (3.46)	1 (2.13)	10 (3.69)		2 (3.17)	9 (3.53)	
Place of birth, n (%)				1.000			0.747
Sichuan	302 (94.97)	45 (95.74)	257 (94.83)		61 (96.83)	241 (94.51)	
Non-Sichuan	16 (5.03)	2 (4.26)	14 (5.17)		2 (3.17)	14 (5.49)	
Newly diagnosis, n (%)				0.746			0.147
Yes	116 (36.48)	16 (34.04)	100 (36.90)		28 (44.44)	88 (34.51)	
No	202 (63.52)	31 (65.96)	171 (63.10)		35 (55.56)	167 (65.49)	
The onset age of TD (year), Mean ± SD	5.82 ± 2.10	5.67 ± 2.02	5.84 ± 2.12	0.722	5.89 ± 1.99	5.80 ± 2.13	0.673
Type of TD, n (%)				0.970			0.711
Provisional tic disorder	167 (52.52)	25 (53.19)	142 (52.40)		31 (49.21)	136 (53.33)	
Chronic tic disorder	126 (39.62)	18 (38.30)	108 (39.85)		28 (44.44)	98 (38.43)	
Tourette syndrome	25 (7.86)	4 (8.51)	21 (7.75)		4 (6.35)	21 (8.24)	
Onset symptom of TD, n (%)				0.583			0.450
Motor tic	164 (51.57)	21 (44.68)	143 (52.77)		32 (50.79)	132 (51.76)	
Vocal tic	20 (6.29)	3 (6.38)	17 (6.27)		6 (9.52)	14 (5.49)	
Both	134 (42.12)	23 (48.94)	111 (40.96)		25 (39.68)	109 (42.75)	
Tic severity (YGTSS), n (%)				0.059			0.193
Severe	10 (3.14)	2 (4.26)	8 (2.95)		1 (1.59)	9 (3.53)	
Moderate	53 (16.67)	13 (27.66)	40 (14.76)		15 (23.81)	38 (14.90)	
Mild	255 (80.19)	32 (68.09)	223 (82.29)		47 (74.60)	208 (81.57)	
Comorbidity, n (%)				0.329			0.466
Yes	203 (63.84)	27 (57.45)	176 (64.94)		43 (68.25)	160 (62.75)	
No	115 (36.16)	20 (42.55)	95 (35.06)		20 (31.75)	95 (37.25)	
Adverse drug reaction, n (%)				0.624			0.189
Yes	281 (88.36)	43 (91.49)	238 (87.82)		59 (93.65)	222 (87.06)	
No	37 (11.64)	4 (8.51)	33 (12.18)		4 (6.35)	33 (12.94)	
Treatment course, n (%)				0.835			0.126
1–3 months	171 (53.77)	23 (48.94)	148 (54.61)		42 (66.67)	129 (50.59)	
4–6 months	61 (19.18)	11 (23.40)	50 (18.45)		8 (12.70)	53 (20.78)	
7–12 months	27 (8.49)	4 (8.51)	23 (8.49)		3 (4.76)	24 (9.41)	
≥ 1 year	59 (18.55)	9 (19.15)	50 (18.45)		10 (15.87)	49 (19.22)	
**Caregivers**							
Age (year), n (%)				0.315			0.772
< 30	33 (10.38)	3 (6.38)	30 (11.07)		5 (7.94)	28 (10.98)	
30–50	249 (78.30)	36 (76.60)	213 (78.60)		50 (79.36)	199 (78.04)	
≥ 50	36 (11.32)	8 (17.02)	28 (10.33)		8 (12.70)	28 (10.98)	
Relationship with child, n (%)				0.242			0.170
Parent	252 (79.25)	34 (72.34)	218 (80.44)		54 (85.71)	198 (77.65)	
Other	66 (20.75)	13 (27.66)	53 (19.56)		9 (14.29)	57 (22.35)	
Family structure, n (%)				1.000			0.026
Married	305 (95.91)	45 (95.74)	260 (95.94)		57 (90.48)	248 (97.25)	
Single-parent	13 (4.09)	2 (4.26)	11 (4.06)		6 (9.52)	7 (2.75)	
Job, n (%)				0.034			0.536
Worker	29 (9.12)	5 (10.64)	24 (8.86)		7 (11.11)	22 (8.63)	
Farmer	78 (24.53)	19 (40.43)	59 (21.77)		20 (31.75)	58 (22.75)	
Administrative leading cadres	46 (14.47)	7 (14.89)	39 (14.39)		7 (11.11)	39 (15.29)	
Specialist or technicist	51 (16.04)	7 (14.89)	44 (16.24)		9 (14.29)	42 (16.47)	
Other	114 (35.85)	9 (19.15)	105 (38.75)		20 (31.75)	94 (36.86)	
Educational background of caregiver, n (%)				0.046			0.330
Junior high school and below	137 (43.08)	28 (59.57)	109 (40.22)		31 (49.20)	106 (41.57)	
Senior high school or technical secondary school	98 (30.82)	11 (23.40)	87 (32.10)		20 (31.75)	78 (30.59)	
Undergraduate and above	83 (26.10)	8 (17.02)	75 (27.68)		12 (19.05)	71 (27.84)	
Residence, n (%)				0.017			0.482
Urban	161 (50.63)	16 (34.04)	145 (53.51)		29 (46.03)	132 (51.76)	
Rural	157 (49.37)	31 (65.96)	126 (46.49)		34 (53.97)	123 (48.24)	
Circumstance, n (%)				0.029			0.886
Noisy	40 (12.58)	11 (23.40)	29 (10.70)		8 (12.70)	32 (12.55)	
Common	155 (48.74)	23 (48.94)	132 (48.71)		29 (46.03)	126 (49.41)	
Quiet	123 (38.68)	13 (27.66)	110 (40.59)		26 (41.27)	97 (38.04)	
Family relationship				0.001			0.111
Harmonious	216 (67.92)	21 (44.68)	195 (71.96)		36 (57.14)	180 (70.59)	
Common	81 (25.47)	19 (40.43)	62 (22.88)		22 (34.92)	59 (23.14)	
Unharmonious	21 (6.60)	7 (14.89)	14 (5.17)		5 (7.94)	16 (6.27)	
Family discipline				0.715			0.924
Strict	79 (24.84)	14 (29.79)	65 (23.99)		15 (23.81)	64 (25.10)	
Common	213 (66.98)	30 (63.83)	183 (67.53)		42 (66.67)	171 (67.06)	
Relax	26 (8.18)	3 (6.38)	23 (8.49)		6 (9.52)	20 (7.84)	
Medical expenses payment				0.379			0.068
Medical insurance of rural corporation	27 (8.49)	7 (14.89)	20 (7.38)		10 (15.87)	17 (6.67)	
Commercial health insurance	11 (3.46)	1 (2.13)	10 (3.69)		3 (4.76)	8 (3.14)	
Social health insurance	41 (12.89)	5 (10.64)	36 (13.28)		5 (7.94)	36 (14.11)	
Self-paying	239 (75.16)	34 (72.34)	205 (75.65)		45 (71.43)	194 (76.08)	

SD: Standard deviation; TD: Tic disorder; YGTSS: Yale global tic severity scale.

### The prevalence of anxiety and depression

In total, 14.78% (47/318) of caregivers among pediatric patients with TD were diagnosed with anxiety, with a mean SAS score of 54.81±5.26; and 19.81% (63/318) were diagnosed with depression, with a mean SDS score of 59.64±5.83. Among these, 7.23% (23/318) of caregivers presented with symptoms of both depression and anxiety (Table 2).

**Table 2:** Prevalence of anxiety and depression.

	n (%)	Score of SAS (Mean ± SD)	n (%)	Score of SDS (Mean ± SD)	Both, n (%)
Total	318 (100)	39.95±9.14	318 (100)	40.42±12.25	/
Normal	271 (85.22)	37.38±6.94	255 (80.19)	35.67±8.05	231 (72.64)
Anxiety/Depression	47 (14.78)	54.81±5.26	63 (19.81)	59.64±5.83	23 (7.23)
Mild	39 (12.26)	52.85±2.48	41 (12.89)	56.25±2.71	/
Moderate	7 (2.20)	63.04±3.13	20 (6.29)	64.88±2.89	/
Severe	1 (0.32)	73.75	2 (0.63)	76.88±6.19	/

SAS: Self-rating anxiety scale; SDS: Self-rating depression scale; SD: Standard deviation.

### Factors associated with anxiety among caregivers

In univariate analysis, caregivers’ job (p = 0.034), education background (p = 0.046), residence (p = 0.017), circumstance (p = 0.029), family relationship (p = 0.001), caregivers’ reaction to the onset of symptom (p = 0.005), evaluation of the services of medical staff (p = 0.034), pediatric patients’ relationship with friends (p = 0.001), self-care ability (p = 0.004), and personality (p = 0.004) were potentially associated with anxiety. In the multivariate logistic model, only the common family relationship (OR = 2.512, p = 0.024), and pediatric patients with unharmonious social relationships (OR = 5.759, p = 0.043) and with introverted personality (OR = 2.402, p = 0.023) remained significant after adjusting for confounders (Table 3).

**Table 3:** Multivariate logistic regression analysis of contributing factors of anxiety.

Variables	β	SE	*p*	OR (95% CI)
Job, n (%)				
Worker	Reference			
Farmer	0.410	0.657	0.533	1.506 (0.415–5.464)
Administrative leading cadres	0.066	0.750	0.930	1.068 (0.246–4.648)
Specialist or technicist	0.449	0.766	0.558	1.566 (0.349–7.029)
Other	-0.356	0.698	0.610	0.701 (0.178–2.752)
Education background of caregiver, n (%)				
Junior high school and below	Reference			
Senior high school or technical secondary school	0.358	0.538	0.506	1.430 (0.498–4.104)
Undergraduate and above	-0.148	0.676	0.827	0.862 (0.229–3.247)
Residence, n (%)				
Urban	Reference			
Rural	0.422	0.472	0.372	1.524 (0.604–3.846)
Circumstance, n (%)				
Noisy	Reference			
Common	-0.604	0.558	0.279	0.547 (0.183–1.632)
Quiet	-0.569	0.617	0.357	0.566 (0.169–1.898)
Family relationship				
Harmonious	Reference			
Common	0.921	0.408	**0.024**	2.512 (1.13–5.585)
Unharmonious	0.664	0.642	0.301	1.942 (0.552–6.831)
Reaction to the onset of symptom				
Scold and curb it	Reference			
Angry but put it aside	0.142	0.561	0.801	1.152 (0.383–3.463)
Ignore and distract attention	-0.171	0.484	0.724	0.843 (0.327–2.175)
Evaluation of the services of medical staff				
Good	Reference			
Moderate	-0.095	0.527	0.857	0.909 (0.324–2.554)
Bad	0.245	0.929	0.792	1.278 (0.207–7.892)
Relationship with friends				
Harmonious	Reference			
Moderate	-0.016	0.386	0.967	0.984 (0.462–2.097)
Unharmonious	1.751	0.863	**0.043**	5.759 (1.06–31.282)
Self-care ability				
Strong	Reference			
Moderate	-0.243	0.422	0.564	0.784 (0.343–1.792)
Weak	0.796	0.573	0.165	2.217 (0.721–6.822)
Personality				
Extraverted	Reference			
Introverted	0.876	0.385	**0.023**	2.402 (1.13–5.103)

β = unstandardized regression coefficient; SE = Standard Error; OR = odds ratio; CI = confidence interval.

### Factors associated with depression among caregivers

In univariate analysis, family structure (p = 0.026), cognition of TD (p = 0.039), cognition of the importance of medication (p = 0.043), cognition of the adverse effect of medication (p = 0.013), numbers of pediatric patients’ friends (p = 0.028) were potentially associated with depression. In the multivariate logistic model, only the single-parent family (OR = 4.805, p = 0.011), mistaken cognition of TD (OR = 0.357, p = 0.031), and pediatric patients ***with*** fewer friends (OR = 3.377, p = 0.006) remained significant after adjusting for confounders (Table 4).

**Table 4:** Multivariate logistic regression analysis of contributing factors of depression.

Variables	β	SE	*p*	OR (95% CI)
Family structure, n (%)				
Married	Reference			
Single-parent	1.570	0.619	**0.011**	4.805 (1.429–16.159)
Cognition of TD				
A disease	Reference			
A harmful behavior habit	-1.029	0.477	**0.031**	0.357 (0.140–0.910)
Whether understanding the importance of medication				
Yes	Reference			
Moderate	-0.503	0.400	0.209	0.605 (0.276–1.326)
No	0.174	0.442	0.693	1.190 (0.501–2.829)
Whether understanding the adverse effect of medication				
Yes	Reference			
Moderate	-0.826	0.445	0.064	0.438 (0.183–1.048)
No	-0.070	0.419	0.867	0.932 (0.41–2.12)
Numbers of friends				
More (≥6)	Reference			
Moderate (3–5)	0.328	0.326	0.314	1.388 (0.733–2.63)
Fewer (<3)	1.217	0.439	**0.006**	3.377 (1.429–7.98)

β = unstandardized regression coefficient; SE = Standard Error; OR = odds ratio; CI = confidence interval.

## Discussion

The findings of this cross-sectional study indicate that the symptoms of anxiety and depression are common among caregivers of pediatric patients with TD in western China. Besides, we identify that family relationship and pediatric patients’ social relationships and personality are related to the anxiety of caregivers, while family structure, cognition of TD, and pediatric patients’ social relationship are related to the depression of caregivers, which points out the direction of comprehensive support providing for these families.

The prevalence of anxiety and depression among caregivers in our study is lower than that found in similar studies in China and other countries. Zhu and colleagues [15] reported that 18.97% and 26.44% of 174 parents of children with TD presented the symptoms of anxiety and depression in Beijing, respectively, which was a little higher than our results (14.78% and 19.81%). It seems like parents from first-tier city afford more parenting stress than those from the western region in China. The medical and economic conditions in different countries can also result in different morbidity. Jalenques et al. [22] conducted a controlled study in France and found that parents of adolescents with TS reported higher levels of anxiety and depression (44.9% and 26.1%); among them, 18.7% of mothers and 7.9% of fathers were taking medications for psychiatric symptoms. It may be related to participants from the same family who were easily affected by their spouses, leading to higher psychiatric morbidity, while our study only assessed the main caregiver of each pediatric patient with TD based on a larger sample size. A previous study related to the psychological burden on caregivers of children with chronic diseases showed that 66.7% of caregivers had anxiety symptoms and 74.5% had depressive symptoms [23], which were much higher than those of children with TD, indicating that the psychological burden derived from TD ranked in the relatively low- and middle-range among chronic diseases.

Multivariate analysis reveals that common family relationship and single-parent family are risk factors for anxiety and depression as compared to harmonious and married ones. In our study, the unharmonious family relationship was no significantly associated with anxiety, which might be because of the less proportion of this group (6.60%, 21/318) in our sample. Due to the complexity of the symptoms and the high parenting stress of TD patients, those caregivers who are single, widowed, divorced, separated, or lack appropriate support from other family members are more likely to experience greater mental burden and increase the risk for psychological illness. As a study reported by Lee et al. [24], family support and encouragement, the sharing of experiences among parents, and intervention by and assistance from society can produce a positive effect on reducing parenting stress, while there were 5.5% to 15.83% of subjects received no functional social support as above. A qualitative study discussed issues relating to the support offered, with many highlighting difficulties in knowing where to get advice and support [25]. The environment lacking social support and services for families with TD would be further tougher and helpless for isolated caregivers and lead to the incidence of anxiety or depression.

A significant negative correlation was found between mistaken cognition of TD and caregivers’ depression, which adversely indicated that those regarding TD as the disease tended to present the symptom of depression. Those who misinterpreted TD as a harmful behavior habit presented fewer depressive symptoms. It may be attributed to the caregivers and pediatric patients undergoing treatment in our hospital who have received correct education on disease cognition from clinicians before the investigation. Given its genetic etiology, parents’ guilt and self-blame may have inhibited them from seeking professional help after learning about the inheritability of the syndrome, and lower self-esteem and the experience of perceived stigma concerning TD may further increase their negative emotions [26].

As tics are the main characteristic of TD, the public stereotypes and social stigma about the disorder bring discriminatory behavior and bullying [27,28]. Our results showed that pediatric patients with introverted personalities, fewer friends, and unharmonious social relationships are important factors contributing to anxiety and depression among caregivers, which supports evidence from previous observations of Jalenques et al. [22]. The constrained relationships with friends and marginalization due to TD impelled the child to become socially isolated, which induced the caregiver to excessively worry about the interpersonal relationship and future life of the pediatric patient. In addition, O‘Hare et al. in a qualitative semi-structured interview study presented that social isolation was a significant contextual factor contributing to parental distress [29]. The stigmatization of the disease is universal but affected by culture and history [30]. The European clinical guideline follows that it is essential to provide psycho-education for the patients, as well as their environment, to the benefit of understanding the condition and releasing mental stress [14].

This study has some limitations. Firstly, we included all pediatric patients with a diagnosis of TD including PTD, CTD, and TS, thus the results were unable to represent anyone among which types. Moreover, this study only investigated caregivers from western China that could not apply to other regions, and some factors had inadequate sample sizes that may render the results instability. Further multi-center research with a larger sample size should be carried out to explore the caregivers of pediatric patients with TD nationwide to obtain a sufficient understanding of the current situation in China as a whole. In addition, cross-sectional studies are hard to the determination of causal inferences, and longitudinal studies need to be conducted to further confirm the reasons underlying this psychiatric morbidity.

## Conclusions

Our study findings provide a better understanding of anxiety and depression among caregivers of pediatric patients with TD, and we explore contributing factors that may be involved. It may have beneficial implications for approaches to providing comprehensive support to these families. Programs that contribute to increasing public understanding and tolerance of TD sufferers are needed. Most importantly, giving more social support to caregivers and families with TD may also facilitate improving well-being and decreasing parenting stress.

## Supporting information

S1 Table The cognition and attitude towards TD and treatment from caregivers’ perspectives. (DOCX)

S2 Table The social relationship and psychological status of pediatric patients with TD. (DOCX)
