# Supplementary material for: Anxiety and depression among caregivers of pediatric patients with tic disorder in western China: A cross-sectional study
Source: PLoS One. 2023 Jul 28;18(7):e0289381. doi: 10.1371/journal.pone.0289381 (PMC10381038; doi:10.1371/journal.pone.0289381)
Supplement: S1 Table — (DOCX) [file pone.0289381.s001.docx]

**S1 Table. The cognition and attitude towards TD and treatment from caregivers’ perspectives**

| **Items, n (%)** | **Total (n=318)** | **Anxiety** | | ***p*** | **Depression** | | ***p*** |
| --- | --- | --- | --- | --- | --- | --- | --- |
|  |  | **Yes (n=47)** | **No (n=271)** |  | **Yes (n=63)** | **No (n=255)** |  |
| **Concerning TD** |  |  |  |  |  |  |  |
| Cognition of TD |  |  |  | 0.683 |  |  | 0.039 |
| A disease | 262 (82.39) | 40 (85.11) | 222 (81.92) |  | 57 (90.48) | 205 (80.39) |  |
| A harmful behavior habit | 56 (17.61) | 7 (14.89) | 49 (18.08) |  | 6 (9.52) | 50 (19.61) |  |
| Reaction to the onset of symptom |  |  |  | 0.005 |  |  | 0.870 |
| Scold and curb it | 81 (25.47) | 18 (38.30) | 63 (23.25) |  | 15 (23.81) | 66 (25.88) |  |
| Angry but put it aside | 35 (11.01) | 9 (19.15) | 26 (9.59) |  | 8 (12.70) | 27 (10.59) |  |
| Ignore and distract attention | 202 (63.52) | 20 (42.55) | 182 (67.16) |  | 40 (63.49) | 162 (63.53) |  |
| Whether believing that the "folk prescription" can cure TD |  |  |  | 0.818 |  |  | 0.320 |
| Yes | 1 (0.31) | 0 (0) | 1 (0.37) |  | 0 (0) | 1 (0.40) |  |
| Probably | 30 (9.43) | 5 (10.64) | 25 (9.23) |  | 9 (14.29) | 21 (8.24) |  |
| No | 287 (90.25) | 42 (89.36) | 245 (90.41) |  | 54 (85.71) | 233 (91.37) |  |
| Whether giving up the medication and try the "folk prescription" |  |  |  | 0.163 |  |  | 0.590 |
| Yes | 46 (14.47) | 11 (23.40) | 35 (12.92) |  | 8 (12.70) | 38 (14.90) |  |
| Probably | 22 (6.92) | 2 (4.26) | 20 (7.38) |  | 6 (9.52) | 16 (6.27) |  |
| No | 250 (78.62) | 34 (72.34) | 216 (79.70) |  | 49 (77.78) | 201 (78.82) |  |
| Whether reviewing TD regularly |  |  |  | 0.157 |  |  | 0.624 |
| Yes | 296 (93.08) | 43 (91.49) | 253 (93.36) |  | 60 (95.24) | 236 (92.55) |  |
| Moderate | 18 (5.66) | 2 (4.26) | 16 (5.90) |  | 2 (3.17) | 16 (6.27) |  |
| No | 4 (1.26) | 2 (4.26) | 2 (0.74) |  | 1 (1.59) | 3 (1.18) |  |
| Whether attaching importance to TD |  |  |  | 0.174 |  |  | 0.307 |
| Yes | 261 (82.08) | 36 (76.60) | 225 (83.03) |  | 54 (85.71) | 207 (81.18) |  |
| Moderate | 42 (13.21) | 10 (21.28) | 32 (11.81) |  | 5 (7.94) | 37 (14.51) |  |
| No | 15 (4.72) | 1 (2.13) | 14 (5.17) |  | 4 (6.35) | 11 (4.31) |  |
| Whether understanding the condition of TD |  |  |  | 0.582 |  |  | 0.950 |
| Yes | 100 (31.45) | 17 (36.17) | 83 (30.63) |  | 19 (30.16) | 81 (31.76) |  |
| Moderate | 145 (45.60) | 18 (38.30) | 127 (46.86) |  | 30 (47.62) | 115 (45.10) |  |
| No | 73 (22.96) | 12 (25.53) | 61 (22.51) |  | 14 (22.22) | 59 (23.14) |  |
| Whether trusting in medical staff |  |  |  | 0.319 |  |  | 0.368 |
| Yes | 300 (94.34) | 43 (91.49) | 257 (94.83) |  | 58 (92.06) | 242 (94.90) |  |
| Moderate | 18 (5.66) | 4 (8.51) | 14 (5.17) |  | 5 (7.94) | 13 (5.10) |  |
| Evaluation of the services of medical staff |  |  |  | 0.034 |  |  | 0.474 |
| Good | 267 (83.96) | 34 (72.34) | 233 (85.98) |  | 55 (87.30) | 212 (83.14) |  |
| Moderate | 43 (13.52) | 10 (21.28) | 33 (12.18) |  | 8 (12.70) | 35 (13.73) |  |
| Bad | 8 (2.52) | 3 (6.38) | 5 (1.85) |  | 0 (0) | 8 (3.14) |  |
| **Concerning treatment** |  |  |  |  |  |  |  |
| The way to get the drug information |  |  |  | 0.071 |  |  | 0.734 |
| Doctor | 277 (87.11) | 37 (78.72) | 240 (88.56) |  | 54 (85.71) | 223 (87.45) |  |
| Nurse | 18 (5.66) | 7 (14.89) | 11 (4.06) |  | 3 (4.76) | 15 (5.88) |  |
| Clinical pharmacist | 9 (2.83) | 1 (2.13) | 8 (2.95) |  | 2 (3.17) | 7 (2.75) |  |
| Staff pharmacist | 2 (0.63) | 0 (0) | 2 (0.74) |  | 0 (0) | 2 (0.78) |  |
| Other | 12 (3.77) | 2 (4.26) | 10 (3.69) |  | 4 (6.35) | 8 (3.14) |  |
| Whether understanding the administration of drugs through  explanation from medical staff |  |  |  | 0.850 |  |  | 0.119 |
| Yes | 217 (68.24) | 31 (65.96) | 186 (68.63) |  | 38 (60.32) | 179 (70.20) |  |
| Moderate | 71 (22.33) | 12 (25.53) | 59 (21.77) |  | 15 (23.81) | 56 (21.96) |  |
| No | 30 (9.43) | 4 (8.51) | 26 (9.59) |  | 10 (15.87) | 20 (7.84) |  |
| The frequency of non-compliance with the medication of the child |  |  |  | 0.526 |  |  | 0.210 |
| Never | 240 (75.47) | 32 (68.09) | 208 (76.75) |  | 43 (68.25) | 197 (77.25) |  |
| Sometimes | 42 (13.21) | 8 (17.02) | 34 (12.55) |  | 12 (19.05) | 30 (11.76) |  |
| Usually | 16 (5.03) | 3 (6.38) | 13 (4.80) |  | 2 (3.17) | 14 (5.49) |  |
| Always | 20 (6.29) | 4 (8.51) | 16 (5.90) |  | 6 (9.52) | 14 (5.49) |  |
| Satisfaction with the effectiveness of the treatment |  |  |  | 0.140 |  |  | 0.420 |
| Yes | 124 (38.99) | 13 (27.66) | 111 (40.96) |  | 26 (41.27) | 98 (38.43) |  |
| Moderate | 173 (54.40) | 29 (61.70) | 144 (53.14) |  | 31 (49.21) | 142 (55.69) |  |
| No | 21 (6.60) | 5 (10.64) | 16 (5.90) |  | 6 (9.52) | 15 (5.88) |  |
| Evaluation of the therapeutic regimen |  |  |  | 0.850 |  |  | 0.850 |
| Simple | 104 (32.70) | 17 (36.17) | 87 (32.10) |  | 19 (30.16) | 85 (33.33) |  |
| Moderate | 200 (62.89) | 28 (59.57) | 172 (63.47) |  | 41 (65.08) | 159 (62.35) |  |
| Complex | 14 (4.4) | 2 (4.26) | 12 (4.43) |  | 3 (4.76) | 11 (4.31) |  |
| Evaluation of the price of drugs |  |  |  | 0.252 |  |  | 0.405 |
| Expensive | 46 (14.47) | 10 (21.28) | 36 (13.28) |  | 6 (9.52) | 40 (15.69) |  |
| Moderate | 212 (66.67) | 31 (65.96) | 181 (66.79) |  | 43 (68.25) | 169 (66.27) |  |
| Inexpensive | 60 (18.87) | 6 (12.77) | 54 (19.93) |  | 14 (22.22) | 46 (18.04) |  |
| Whether know more information about the medication |  |  |  | 0.312 |  |  | 0.080 |
| Yes | 76 (23.90) | 7 (14.89) | 69 (25.46) |  | 13 (20.63) | 63 (24.71) |  |
| Moderate | 143 (44.97) | 24 (51.06) | 119 (43.91) |  | 23 (36.51) | 120 (47.06) |  |
| No | 99 (31.13) | 16 (34.04) | 83 (30.63) |  | 27 (42.86) | 72 (28.24) |  |
| Whether understanding the importance of medication |  |  |  | 0.361 |  |  | 0.043 |
| Yes | 93 (29.25) | 16 (34.04) | 77 (28.41) |  | 21 (33.33) | 72 (28.24) |  |
| Moderate | 139 (43.71) | 16 (34.04) | 123 (45.39) |  | 19 (30.16) | 120 (47.06) |  |
| No | 86 (27.04) | 15 (31.91) | 71 (26.20) |  | 23 (36.51) | 63 (24.71) |  |
| Whether understanding the adverse effect of medication |  |  |  | 0.130 |  |  | 0.013 |
| Yes | 83 (26.10) | 12 (25.53) | 71 (26.20) |  | 20 (31.75) | 63 (24.71) |  |
| Moderate | 111 (34.91) | 11 (23.40) | 100 (36.90) |  | 12 (19.05) | 99 (38.82) |  |
| No | 124 (38.99) | 24 (51.06) | 100 (36.90) |  | 31 (49.21) | 93 (36.47) |  |
| Whether understanding the harm of medication |  |  |  | 0.856 |  |  | 0.082 |
| Yes | 60 (18.87) | 8 (17.02) | 52 (19.19) |  | 13 (20.63) | 47 (18.43) |  |
| Moderate | 134 (42.14) | 19 (40.43) | 115 (42.44) |  | 19 (30.16) | 115 (45.10) |  |
| No | 124 (38.99) | 20 (42.55) | 104 (38.38) |  | 31 (49.21) | 93 (36.47) |  |
| Whether understanding the harm of non-compliance with medication |  |  |  | 0.364 |  |  | 0.257 |
| Yes | 79 (24.84) | 14 (29.79) | 65 (23.99) |  | 14 (22.22) | 65 (25.49) |  |
| Moderate | 116 (36.48) | 13 (27.66) | 103 (38.01) |  | 19 (30.16) | 97 (38.04) |  |
| No | 123 (38.68) | 20 (42.55) | 103 (38.01) |  | 30 (47.62) | 93 (36.47) |  |
| Whether worrying about the medication addiction |  |  |  | 0.070 |  |  | 0.462 |
| Yes | 174 (54.72) | 33 (70.21) | 141 (52.03) |  | 37 (58.73) | 137 (53.73) |  |
| Uncertain | 96 (30.19) | 9 (19.15) | 87 (32.10) |  | 15 (23.81) | 81 (31.76) |  |
| No | 48 (15.09) | 5 (10.64) | 43 (15.87) |  | 11 (17.46) | 37 (14.51) |  |
