# Supplementary material for: Anxiety and depression among caregivers of pediatric patients with tic disorder in western China: A cross-sectional study
Source: PLoS One. 2023 Jul 28;18(7):e0289381. doi: 10.1371/journal.pone.0289381 (PMC10381038; doi:10.1371/journal.pone.0289381)
Supplement: S2 Table — (DOCX) [file pone.0289381.s002.docx]

**S2 Table. The social relationship and psychological status of pediatric patients with TD**

| **Items, n (%)** | **Total (n=318)** | **Anxiety** | | ***p*** | **Depression** | | ***p*** |
| --- | --- | --- | --- | --- | --- | --- | --- |
|  |  | **Yes (n=47)** | **No (n=271)** |  | **Yes (n=63)** | **No (n=255)** |  |
| Numbers of friends |  |  |  | 0.198 |  |  | 0.028 |
| More (≥6) | 141 (44.34) | 19 (40.43) | 122 (45.02) |  | 23 (36.51) | 118 (46.27) |  |
| Moderate (3-5) | 141 (44.34) | 19 (40.43) | 122 (45.02) |  | 27 (42.86) | 114 (44.71) |  |
| Fewer (<3) | 36 (11.32) | 9 (19.15) | 27 (9.96) |  | 13 (20.63) | 23 (9.02) |  |
| Relationship with friends |  |  |  | 0.001 |  |  | 0.528 |
| Harmonious | 193 (60.70) | 21 (44.68) | 172 (63.47) |  | 36 (57.14) | 157 (61.57) |  |
| Moderate | 115 (36.16) | 20 (42.55) | 95 (35.06) |  | 24 (38.10) | 91 (35.69) |  |
| Unharmonious | 10 (3.14) | 6 (12.77) | 4 (1.48) |  | 3 (4.76) | 7 (2.74) |  |
| Academic performance |  |  |  | 0.142 |  |  | 0.759 |
| Good | 105 (33.02) | 16 (34.04) | 89 (32.84) |  | 21 (33.33) | 84 (32.94) |  |
| Moderate | 193 (60.69) | 25 (53.19) | 168 (61.99) |  | 37 (58.73) | 156 (61.18) |  |
| Bad | 20 (6.29) | 6 (12.77) | 14 (5.17) |  | 5 (7.94) | 15 (5.88) |  |
| Self-care ability |  |  |  | 0.004 |  |  | 0.416 |
| Strong | 87 (27.36) | 14 (29.79) | 74 (26.94) |  | 15 (23.81) | 72 (28.24) |  |
| Moderate | 199 (62.58) | 22 (46.81) | 177 (65.31) |  | 39 (61.90) | 160 (62.75) |  |
| Weak | 32 (10.06) | 11 (23.40) | 21 (7.75) |  | 9 (14.29) | 23 (9.01) |  |
| Interests and hobbies |  |  |  | 0.061 |  |  | 0.888 |
| Extensive | 116 (36.48) | 18 (38.30) | 98 (36.16) |  | 24 (38.10) | 92 (36.08) |  |
| Moderate | 175 (55.03) | 21 (44.68) | 154 (56.83) |  | 33 (52.38) | 142 (55.69) |  |
| Fewer | 27 (8.49) | 8 (17.02) | 19 (7.01) |  | 6 (9.52) | 21 (8.23) |  |
| Temper |  |  |  | 0.103 |  |  | 0.057 |
| Fractious | 144 (45.28) | 28 (59.57) | 116 (42.80) |  | 37 (58.73) | 107 (41.96) |  |
| Common | 143 (44.97) | 15 (31.91) | 128 (47.23) |  | 21 (33.33) | 122 (47.84) |  |
| Soft | 31 (9.75) | 4 (8.51) | 27 (9.96) |  | 5 (7.94) | 26 (10.20) |  |
| Personality |  |  |  | 0.004 |  |  | 0.120 |
| Extraverted | 171 (53.77) | 16 (34.04) | 155 (57.20) |  | 28 (44.44) | 143 (56.08) |  |
| Introverted | 147 (46.23) | 31 (65.96) | 116 (42.80) |  | 35 (55.56) | 112 (43.92) |  |
